# Crystal structure of a short-chain dehydrogenase from *Brucella ovis* with apo and coenzyme NAD^+^-bound protomer chains

**DOI:** 10.1107/S2053230X25009227

**Published:** 2025-11-11

**Authors:** Sean P. Zupko, Amelia T. Konstanty, Steve J. Mayclin, Ryan Choi, Dmitry Serbzhinskiy, Emily Robles, Victoria Moses, Lynn K. Barrett, Wesley C. Van Voorhis, Tom E. Edwards, Peter J. Myler, Andrew T. Torelli, Jarrod B. French, Katherine A. Hicks

**Affiliations:** a Department of Chemistry State University of New York at Cortland Cortland NY13045 USA; b UCB BioSciences, Bainbridge Island, WA98110, USA; c Seattle Structural Genomics Center for Infectious Disease (SSGCID), Seattle, WA98105, USA; d Division of Allergy and Infectious Diseases, Department of Medicine University of Washington Seattle WA98195 USA; e Allegheny College, Meadville, PA16335, USA; f Jacksonville State University Jacksonville AL36265 USA; g Center for Global Infectious Disease Research Seattle Children’s Research Institute Seattle WA98105 USA; h Department of Chemistry Ithaca College Ithaca NY14850 USA; i The Hormel Institute University of Minnesota Austin MN55912 USA; Bristol-Myers Squibb, USA

**Keywords:** short-chain dehydrogenase/reductases, research experience for undergraduates, redox chemistry, coenzyme binding, SSGCID

## Abstract

Short-chain dehydrogenases (SDRs) are a family of NAD(P)-dependent enzymes involved in redox reactions, specifically carbonyl–alcohol reductions. Here, we report the apo and NAD^+^-bound structures of an SDR from the pathogenic organism *Brucella ovis*. *B. ovis* primarily affects sheep and other livestock, resulting in reduced fertility. Based on sequence and structural alignment, the *B. ovis* SDR (BoSDR) is a classical SDR. Classical SDRs have a canonical Y*xxx*K active-site sequence in which the catalytic general base is a tyrosine residue located at position 163. In addition, the putative active site also contains a serine residue (Ser150) and lysine residue (Lys167) that are hypothesized to be involved in catalysis. BoSDR is a biological and crystallographic tetramer. In the coenzyme-bound structure, two different orientations of the NAD^+^ coenzyme are fortuitously observed, which provides insights into the conformational changes that accompany coenzyme binding. The apo and NAD^+^-bound structures provide valuable information about the unique structural features of enzymes in the SDR superfamily.

## Abstract 2

This article describes the structural characterization of a short-chain dehydrogenase from the pathogenic bacterium *Brucella ovis*. In this manuscript, two structures are described: the structure of the apo protein and a structure containing the coenzyme NAD^+^.

## 1. Introduction

*Brucella ovis* is a Gram-negative bacterium that often circulates in sheep and has been found to cause reduced fertility in rams (Brunno Soares Oliveira *et al.*, 2024 ▸). It primarily targets males, where they can become persistently infected and can transmit the infection to other males. Other animals, including goats, bighorn sheep, white-tailed deer and pregnant cows, have also reportedly been infected (Brunno Soares Oliveira *et al.*, 2024 ▸; Olsen & Palmer, 2014 ▸).

**Figure 1:**
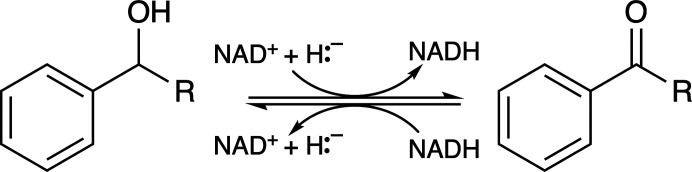
SDR-catalyzed reaction. The substrate(s) for BoSDR are unknown. However, these enzymes are short-chain dehydrogenases (SDRs), which are NAD(P)-dependent oxidoreductase enzymes. These enzymes are involved in both the oxidation of alcohols (example reaction shown above with *R* referring to the rest of the molecule) and the reduction of ketones, often with broad substrate specificities (Roth *et al.*, 2018 ▸; Wu *et al.*, 2007 ▸; Asada *et al.*, 2009 ▸).

In this paper, we describe and discuss the crystal structure of a short-chain dehydrogenase reductase (SDR) from *B. ovis* (BoSDR; PDB entries 5ha5 and 5er6). SDRs are a large family of NAD(P)-dependent enzymes that can perform a wide variety of applications, including carbonyl–alcohol oxido­reductions (Roth *et al.*, 2018 ▸). SDRs are an important family of enzymes, as they have critical roles in lipid, amino-acid, carbohydrate, cofactor, hormone and xenobiotic metabolism (Roth *et al.*, 2018 ▸; Oppermann *et al.*, 2003 ▸). Most commonly, the mechanism involves a hydride and proton transfer involving NAD(P) and a tyrosine residue in the active site (Fig. 1 ▸).

Due to the broad applications of SDRs, they have received attention as biocatalysts (Shanbhag, 2023 ▸). For example, SDRs can reduce alkenes and carbonyls, which increases their possible use in organic synthesis (Roth *et al.*, 2018 ▸). They have also been studied using *in silico* screening methods (Beck *et al.*, 2017 ▸) and multi-disciplinary biological approaches (Qian *et al.*, 2024 ▸). This work highlights the importance of SDRs as potential drug-development targets and the benefits of using structure-guided approaches to inform function as well as a basis for inhibitor design.

Here, we report the apo and NAD^+^-bound structures of BoSDR, a classical SDR. BoSDR contains a conserved, catalytic tyrosine residue, Tyr163 in this structure, which is part of the canonical Y*xxx*K sequence that is commonly seen in the active site of classical SDRs (Man *et al.*, 2015 ▸). Together, the apo BoSDR and BoSDR–NAD^+^ structures provide insights into this SDR enzyme class, including possible conformational changes of the NAD^+^ coenzyme.

## 2. Materials and methods

### 2.1. Macromolecule production

Cloning, expression and purification followed standard protocols as described previously (Bryan *et al.*, 2011 ▸; Choi *et al.*, 2011 ▸; Serbzhinskiy *et al.*, 2015 ▸). The full-length gene, encoding amino acids 1–250, for this short-chain dehydro­genase/reductase-family oxidoreductase from *B. ovis* ATCC 25840 (UniProt A0A0H3ATY4) was PCR-amplified from genomic DNA using the primers shown below in Table 1 ▸. The gene was ligation-independently cloned (LIC) into pBG1861 (Alexandrov *et al.*, 2004 ▸), encoding a noncleavable N-terminal 6×His-tag. Plasmid DNA was transformed into chemically competent cells. The plasmid containing His-tagged BoSDR was expression-tested, and 2 l of culture was grown using auto-induction medium (Studier, 2005 ▸) in a LEX Bioreactor (Epiphyte Three) as described previously (Serbzhinskiy *et al.*, 2015 ▸). The expression clone BrovA.00010.a.B1.GE38036 is available at https://targetstatus.ssgcid.org/Target/BrovA.00010.a.

His-tagged BoSDR was purified in a two-step protocol consisting of an immobilized metal (Ni^2+^)-affinity chromatography (IMAC) step and size-exclusion chromatography (SEC). All chromatography runs were performed on an ÄKTApurifier 10 (GE Healthcare) using automated IMAC and SEC programs (Bryan *et al.*, 2011 ▸). Thawed bacterial pellets (∼25 g) were lysed by sonication in 200 ml buffer consisting of 25 m*M* HEPES pH 7.0, 500 m*M* NaCl, 5%(*v*/*v*) glycerol, 0.5%(*w*/*v*) CHAPS, 30 m*M* imidazole, 10 m*M* MgCl_2_, 1 m*M* TCEP, 250 µg ml^−1^ AEBSF, 0.025%(*w*/*v*) sodium azide. After sonication, the crude lysate was clarified with 20 ml (25 units µl^−1^) of Benzonase and incubated while mixing at room temperature for 45 min. The lysate was clarified by centrifugation at 14 199*g* for 1 h at 4°C using a Sorvall RC5 centrifuge with an F14 rotor (Thermo Scientific). The clarified supernatant was then passed over an Ni–NTA HisTrap FF 5 ml column (GE Healthcare) which was pre-equilibrated with loading buffer composed of 25 m*M* HEPES pH 7.0, 500 m*M* NaCl, 5%(*v*/*v*) glycerol, 30 m*M* imidazole, 1 m*M* TCEP, 0.025%(*w*/*v*) sodium azide. The column was washed with 20 column volumes (CV) of loading buffer and was eluted with loading buffer plus 250 m*M* imidazole in a linear gradient over 7 CV. Peak fractions were pooled and concentrated to 5 ml. A SEC column (Superdex 75, GE Healthcare) was equilibrated with running buffer composed of 20 m*M* HEPES pH 7.0, 300 m*M* NaCl, 5%(*v*/*v*) glycerol, 1 m*M* TCEP. The peak fractions were collected and analyzed using SDS–PAGE for the protein of interest. The protein eluted as a mostly single/monodisperse peak, with >75% of the protein product in the molecular-mass range of approximately 55 kDa (expected molecular weight 25 kDa). The peak fraction was pooled and concentrated to 74.3 mg ml^−1^ using an Amicon centrifugal concentrator (Millipore). Aliquots of 110 µl were flash-frozen in liquid nitrogen and stored at −80°C until use.

### 2.2. Crystallization, data collection and processing

Crystals of BoSDR in the presence of NAD^+^ (BoSDR–NAD^+^) and the apo protein (apo BoSDR) were obtained as described in Table 2 ▸. Data were integrated using *XDS* and were scaled using *XSCALE* (Kabsch, 2010 ▸). Intensities were converted to amplitudes using the *TRUNCATE* utility (French & Wilson, 1978 ▸) within the *CCP*4 suite (Agirre *et al.*, 2023 ▸). Additional data-collection information is included in Table 3 ▸. The raw data for PDB entries 5ha5 and 5er6 are available at https://proteindiffraction.org/search/?q=5ha5 and https://proteindiffraction.org/search/?q=5er6, respectively.

### 2.3. Structure solution and refinement

The structure of BoSDR was determined by molecular replacement using *S*-2-hydroxypropyl coenzyme M dehydro­genase from *Xanthobacter autotrophicus* Py2 (PDB entry 4gh5; Bakelar *et al.*, 2013 ▸) as a starting model. Molecular replacement was performed using *MOLREP* (Vagin & Teplyakov, 2010 ▸) within the *CCP*4 suite (Agirre *et al.*, 2023 ▸). The model was improved through rounds of refinement using *Phenix* (Liebschner *et al.*, 2019 ▸) and manual model building with *Coot* (Emsley *et al.*, 2010 ▸). Refinement statistics are provided in Table 4 ▸. The BoSDR–NAD^+^ structure was deposited in the Protein Data Bank as PDB entry 5ha5 and the apo BoSDR structure was deposited as PDB entry 5er6. All structural figures were made using *PyMOL* (*The PyMOL Molecular Graphics System*, version 3.0; Schrödinger) and chemical structures were made using *CHEMDRAW* version 20.1.

## 3. Results and discussion

This *B. ovis* SDR (BoSDR; PDB entry 5ha5) crystallized in the monoclinic space group *P*12_1_1 with four molecules in the asymmetric unit. This is consistent with the proposed tetrameric biological unit based on *PISA* analysis (Krissinel & Henrick, 2007 ▸). The crystals have a solvent content of 52% with a Matthews coefficient of 2.54 Å^3^ Da^−1^. Based on sequence similarity, *S*-2-hydroxypropyl coenzyme M dehydrogenase from *X. autotrophicus* Py2 (38% sequence identity; PDB entry 4gh5; Bakelar *et al.*, 2013 ▸) was used as the starting model for molecular replacement.

**Figure 2:**
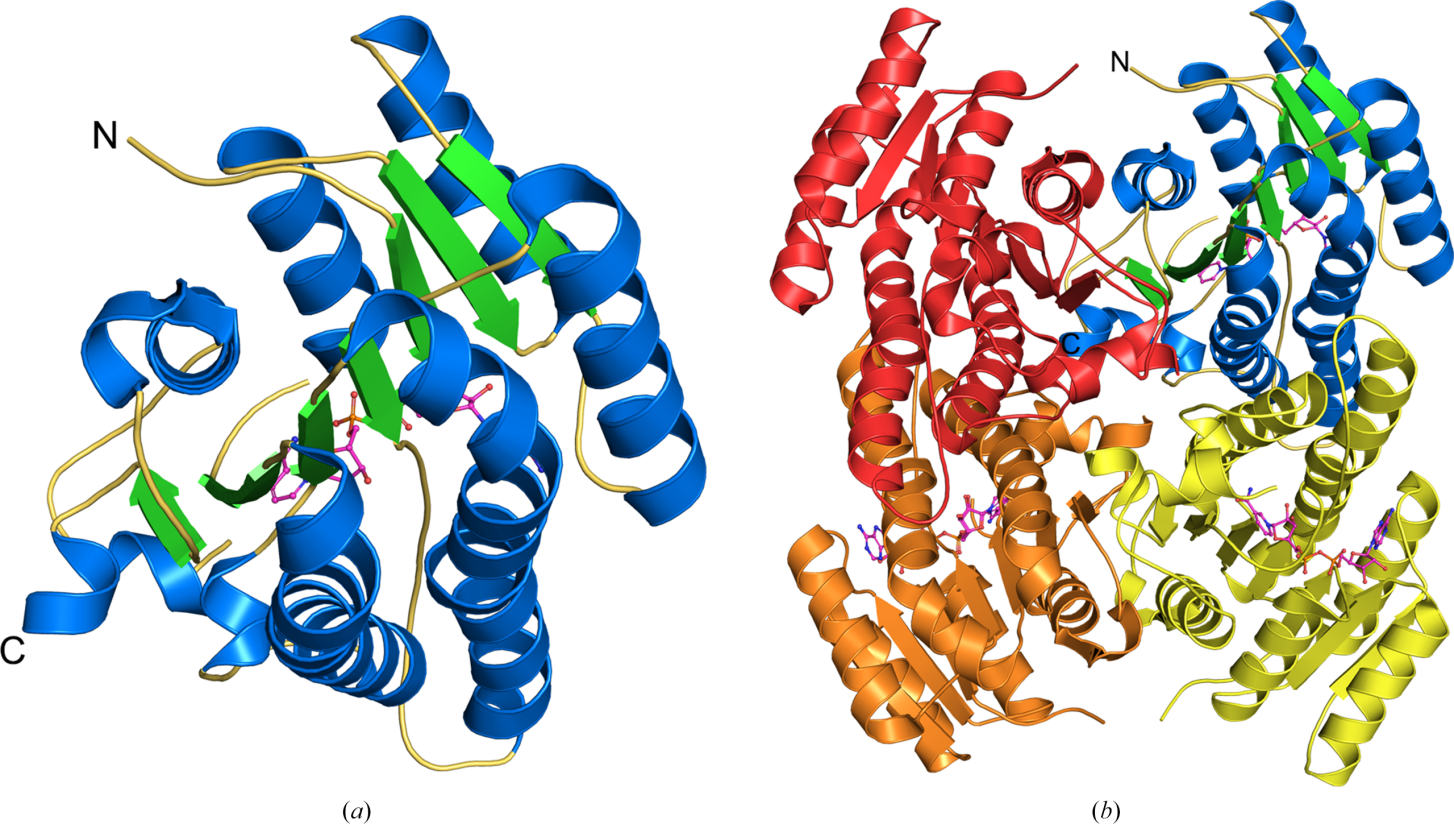
Overall structure of BoSDR. (*a*) Like other SDRs, the protomer of BoSDR has a central β-sheet flanked by α-helices. The NAD^+^ coenzyme is shown in ball-and-stick representation with purple C atoms. Other heteroatoms are shown using standard coloring. Here, β-sheets are shown in green, α-helices are shown in blue and loops are in yellow. (*b*) The overall structure of BoSDR is tetrameric. Chain *A* is colored red, chain *B* is orange, chain *C* is colored by secondary structure as in (*a*) and chain *D* is yellow. The NAD^+^ is shown in ball-and-stick representation with the coloring as in (*a*).

Like other members of the SDR family (Belfon *et al.*, 2024 ▸; Tanaka *et al.*, 2001 ▸), BoSDR has a Rossmann fold with a central parallel β-sheet with α-helices on both sides (Fig. 2 ▸*a*). The overall structure is a tetramer with a molecular mass for the biological complex of 105.7 kDa (Fig. 2 ▸*b*), consistent with analysis by *PDBePISA*, which calculates a tetrameric complex with a total buried surface area of 1570 Å^2^ (Krissinel & Henrick, 2007 ▸). Analysis by size-exclusion chromatography (SEC) shows a monodisperse sample, although the predicted molecular weight is closer to that expected for a dimer. The structure (Fig. 2 ▸*b*), however, is clearly tetrameric with a large buried surface area, as described above. Like BoSDR, the SDRs are often dimeric or tetrameric, which is consistent with the assigned quaternary structure for BoSDR (Kavanagh *et al.*, 2008 ▸). A topology map (Supplementary Fig. S1) is provided to highlight the arrangement of the α-helices and β-sheets.

**Figure 3:**
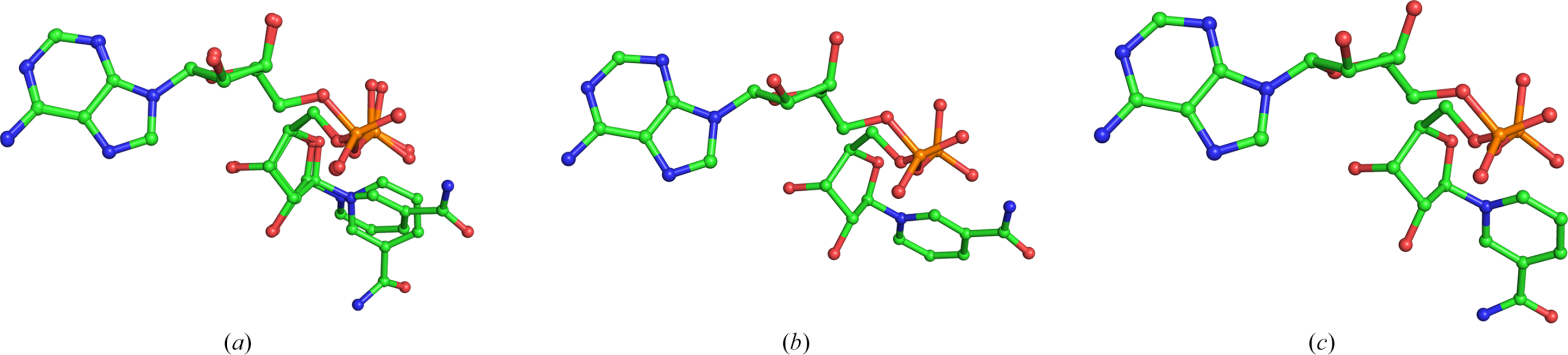
Comparison of NAD^+^ binding. The NAD^+^ coenzyme is depicted in ball-and-stick representation. The C atoms are shown in green. Other heteroatoms are shown using standard coloring. (*a*) Chain *B* contains an NAD^+^ coenzyme modeled with two alternative conformations: *syn* and *anti*. (*b*) The NAD^+^ coenzyme in chain *C* adopts a *syn* conformation. (*c*) By comparison, the NAD^+^ coenzyme in chain *D* adopts an *anti* conformation. Chain *A* does not contain a coenzyme and is not shown

BoSDR has been captured crystallographically both with bound NAD^+^ coenzyme (BoSDR–NAD^+^; PDB entry 5ha5) and in an apo form (apo BoSDR; PDB entry 5er6) that does not contain bound nicotinamide coenzymes. While the protomers all align well (r.m.s.d.s for alignment of chains *B*, *C* and *D* with chain *A* are each 0.1 Å), the BoSDR protomer chains differ in the presence or conformation of bound nicotinamide coenzymes (Fig. 3 ▸). NAD^+^ is not present in chain *A*, while the NAD^+^ coenzyme in chain *B* is present in both the *anti* conformation (modeled with 40% occupancy) and the *syn*conformation (modeled with 60% occupancy). By comparison, the NAD^+^ in chain *C* is modeled with 100% occupancy in the *syn* conformation. Finally, the NAD^+^ coenzyme in chain *D* is modeled with 90% occupancy in the *anti* conformation.

The *syn* conformation of the NAD^+^ coenzyme appears to be stabilized by several hydrogen bonds (Supplementary Fig. S2). The proximal coenzyme phosphate group is positioned 2.7 Å from the nicotinamide N atom, which presumably serves as the hydrogen-bond donor. In addition, the side chain of Ser198 is situated 2.7 Å from the same proximal phosphate group and 2.5 Å from the nicotinamide amine group. Finally, the nicotinamide carbonyl O atom is positioned 2.7 Å from the backbone amine group belonging to Met196. By comparison, the *anti* conformation of the NAD^+^ coenzyme is stabilized by simultaneous hydrogen bonds from the nicotinamide amine group and two conserved active-site residues, Tyr163 and Ser150. In other dehydrogenase enzymes, previous work has observed the nicotinamide cofactor binding in both *syn* and *anti* conformations; however, it has been suggested that the *syn* conformation may be less catalytically competent (Vincent *et al.*, 1997 ▸).

In addition, imidazole is present in all four protomer chains and 1,2-ethanediol is present in chains *A*–*C*. These molecules are likely to be artifacts from either protein purification, crystallization or cryoprotection. Some of the 1,2-ethanediol molecules are near the tetrameric interface, while the imidazole is located near the exterior of the protein (Supplementary Fig. S3).

**Figure 4:**
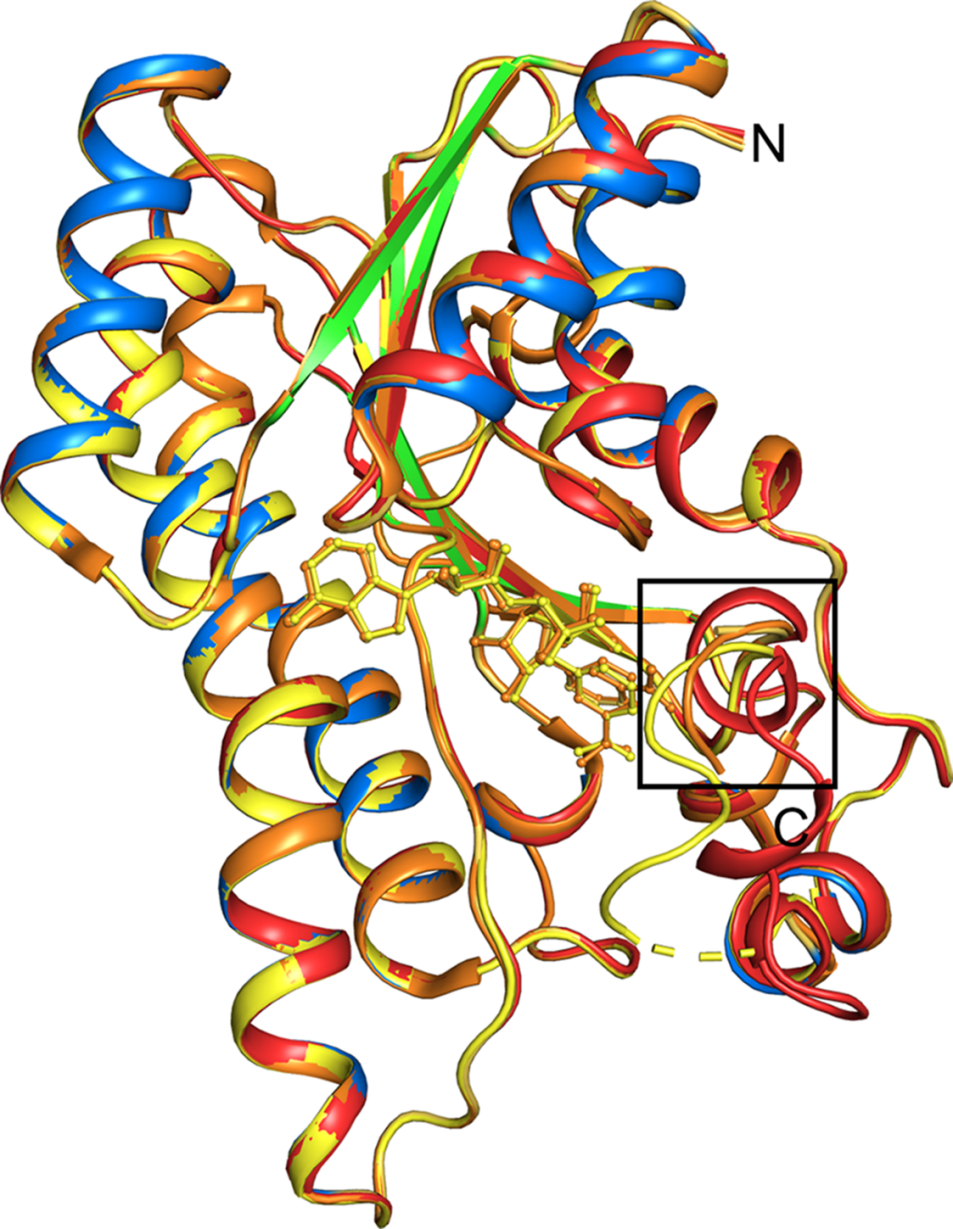
Structural comparison of BoSDR–NAD^+^ protomer chains. The coloring is the same as in Fig. 2 ▸(*b*). The NAD^+^ coenzyme is shown in ball-and-stick representation colored corresponding to its chain. Note that most of the backbone structures overlay closely except for the region around the nicotinamide portion of NAD^+^ (boxed area), which becomes disordered in chains *B*, *C* and *D*. For clarity, the imidazole and 1,2-ethanediol ligands are not depicted.

The four chains of BoSDR have a similar structure (Fig. 4 ▸) except for the area around the nicotinamide ring of the NAD^+^ coenzyme, which is boxed. The viewpoint in Fig. 4 ▸ has been rotated approximately 180° from that in Fig. 2 ▸ to highlight the position of the nicotinamide ring of NAD^+^. Upon NAD^+^ binding (chains *B*, *C* and *D*), this region becomes a disordered loop consisting of residues 198–210. In the absence of NAD^+^ (chain *A*) it is an ordered, small α-helix, and the side chain of Glu199 occupies the space where the nicotinamide ring of the NAD^+^ coenzyme binds. This is the area of the enzyme where the hydride is transferred to the substrate, so it is not surprising that this area would undergo rearrangement upon coenzyme binding (Cho *et al.*, 2005 ▸). Neither BoSDR–NAD^+^ nor apo BoSDR (PDB entries 5ha5 and 5er6, respectively) were crystallized in the presence of a substrate analog nor do they have a ligand bound at the putative active site.

**Figure 5:**
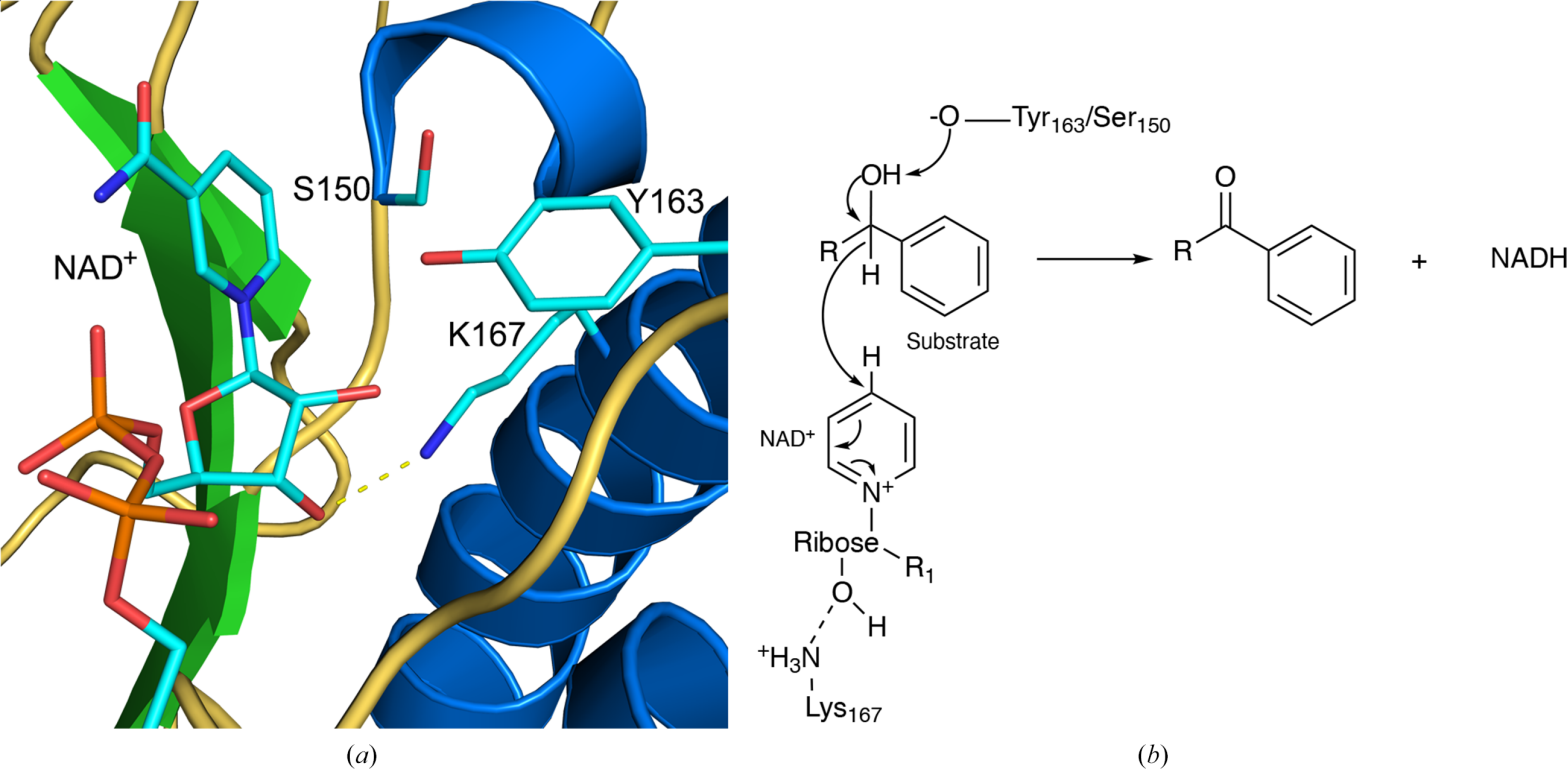
Putative active site of BoSDR. (*a*) The putative active site of BoSDR is depicted with the secondary structure colored as in Fig. 2 ▸(*a*). For clarity, only the active site of chain *C* is shown. C atoms are shown in cyan. Other heteroatoms are shown using standard coloring. For clarity, water molecules are not shown. A hydrogen bond (3.0 Å) between Lys167 and the 3′-OH of NAD^+^ is depicted. (*b*) A potential mechanism for the BoSDR-catalyzed reaction is shown based on the active-site architecture and the mechanism of other classical SDRs (Filling *et al.*, 2002 ▸). For clarity, only the oxidation of an alcohol is shown. Here, *R* refers to the rest of the substrate molecule and *R*_1_ to the rest of the NAD^+^ coenzyme molecule.

Based on sequence homology (Supplementary Fig. S4) we can deduce that the active site of this enzyme is like that of other classical SDRs, with the canonical Y*xxx*K sequence. In this case, the tyrosine residue is located at position 163 and the lysine is at position 167 (Fig. 5 ▸*a*). Moreover, a serine residue (Ser150) is positioned to be involved in catalysis as seen for other SDRs (Filling *et al.*, 2002 ▸). This protein also has the classical NAD^+^-binding motif consisting of the TG*xxx*G*x*G motif (Lesk, 1995 ▸). For clarity, the putative active site for only chain *C* is shown in Fig. 5 ▸. In the absence of NAD^+^ (PDB entry 5er6), the side chains of Tyr163, Lys167 and Ser150 do not change conformation from their orientations in the coenzyme-bound structure (not shown).

In the putative active site (Fig. 5 ▸*a*), the catalytic tyrosine residue is located 4.3 Å from the NAD^+^ carbon involved in hydride transfer. In addition to Tyr163, a serine residue (Ser150) is also conserved (Supplementary Fig. S4) and is within 5.6 Å of the NAD^+^ hydride and within 4.3 Å of the closest C atom of the nicotinamide ring. As has been seen for other SDRs (Filling *et al.*, 2002 ▸), it is possible that either the tyrosine or serine stabilizes the developing negative charge on the oxoanion, although it is more likely that the tyrosine is the proton acceptor, as has been observed for other SDRs (Kavanagh *et al.*, 2008 ▸). To function as a proton acceptor, Tyr163/Ser150 must be present in a deprotonated state. These residue(s) could be deprotonated by a water molecule, the substrate or an unknown general base. In addition, a conserved lysine (Lys167; Supplementary Figs. S4 and S5) is likely to be involved in stabilizing the hydroxyls on the NAD^+^ ribose ring which is located 3.0 Å from the 2′-OH and 3.1 Å from the 3′-OH. Together, this allows us to propose a mechanism for BoSDR consistent with the structural data (Fig. 5 ▸*b*), which can be further characterized and elucidated by future kinetic analyses. In this potential mechanism, Tyr163 deprotonates the substrate alcohol, which is followed by hydride transfer to the NAD^+^ coenzyme. This results in the oxidation of the substrate alcohol to a ketone and the reduction of the NAD^+^ coenzyme to NADH.

Structural analysis suggests that BoSDR is specific for NAD^+^/NADH, rather than NADP^+^/NADPH, as the adenine nucleotide ribose hydroxyls at positions 2′ and 3′ are within hydrogen-bonding distance of only the side chain of Asp45 in chains *B*–*D* (Supplementary Fig. S5). The distances between the Asp45 carboxylic acid group and the coenzyme hydroxyl groups in chains *B* and *C* are identical (2.5 Å to 2′-OH/3′-OH) and slightly longer in chain *D* (2.5 Å to 2′-OH and 2.9 Å to 3′-OH). If BoSDR could utilize an NADP^+^/NADPH coenzyme, it is expected that an arginine residue near the adenine nucleotide ribose would stabilize the densely negatively charged 2′-phosphate group (Belfon *et al.*, 2024 ▸). While an arginine residue (Arg24) is near the coenzyme-binding site, it is positioned away from the ribose towards the exterior of the protein. It is also located 5.4 Å from the closest hydroxyl group on the adenine nucleotide ribose. In addition, an isoleucine residue (Ile45) is partially blocking the site where the 2′-phosphate group would bind. Therefore, it does not seem, based on structural analysis, that BoSDR uses NADP^+^/NADPH as a coenzyme.

**Figure 6:**
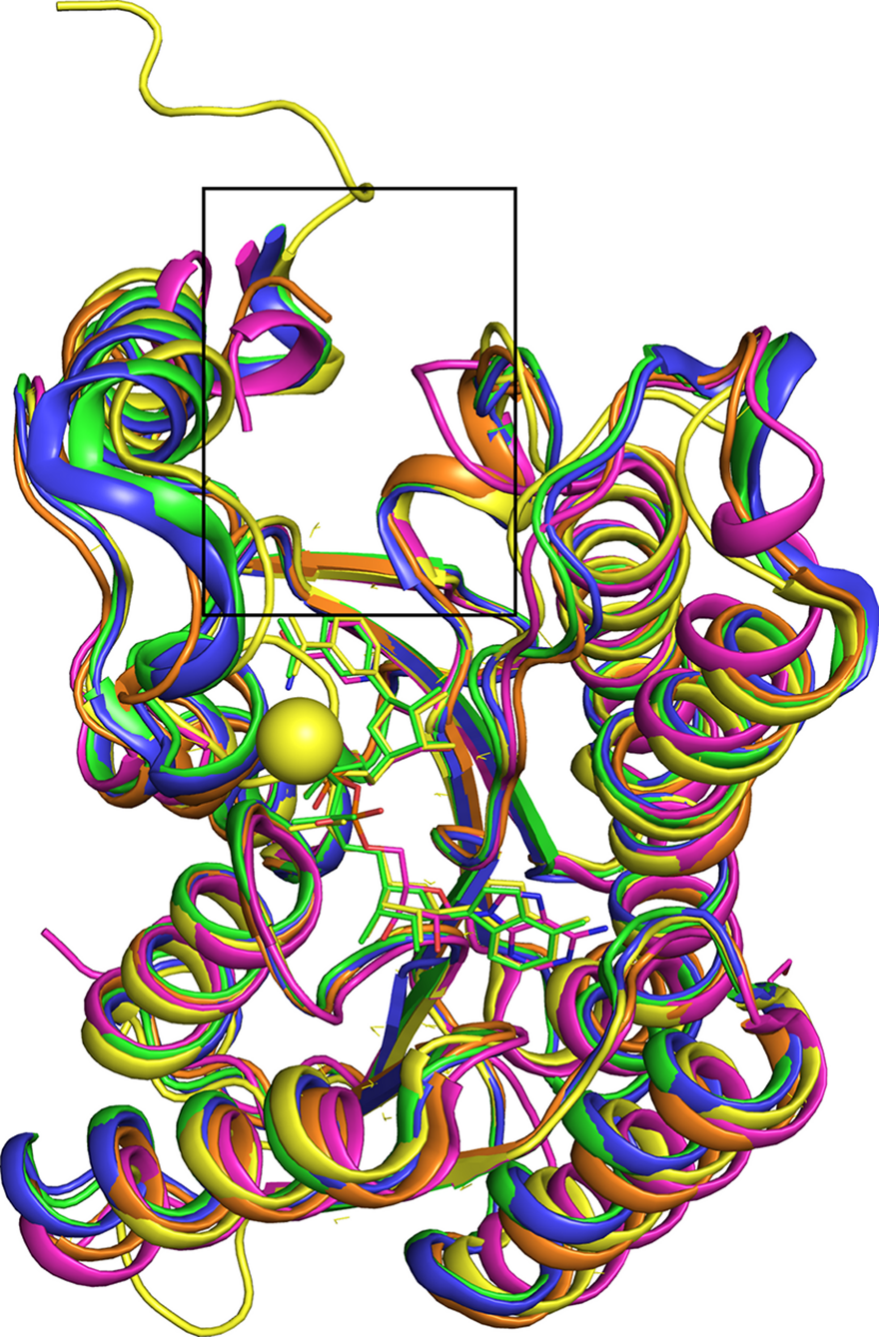
Structural alignment of BoSDR with its closest homologues. The structures are shown in cartoon representation colored by structure. Any bound ions or small molecules are shown in ball-and-stick representation with the same coloring as the protein structure. BoSDR is shown in pink, its closest structural homologue FabG (PDB entry 4nbv) is shown in orange and 2,3-butanediol dehydrogenase (PDB entry 6vsp) is shown in yellow, including a bound sodium cation. The ketoreductase from *Sphingobacterium siyangense* SY1 (PDB entry 8y83) is shown in green and the ketone reductase ChKRED20 from *Chryseobacterium* sp. CA49 is shown in blue (PDB entry 6ixm). For clarity, only a single protomer of each protein is depicted. The cavity leading to the active site is highlighted with a box.

The proteins with the highest structural similarity to BoSDR are shown in Supplementary Table S1 based on *DALI* server results from June 2025 (Holm, 2022 ▸). These proteins include other members of the SDR superfamily. A structural superposition was performed on the top four structural homologues of BoSDR (Fig. 6 ▸). This view is rotated approximately 180° relative to Fig. 2 ▸ to highlight the structural differences. These proteins include *Cupriavidus taiwanensis* FabG (Javidpour *et al.*, 2014 ▸), *Serratia marcescens* 2,3-butanediol dehydrogenase (Subramanian *et al.*, 2020 ▸), *Sphingobacterium siyangense* SY1 ketoreductase (Che *et al.*, 2024 ▸) and the ketone reductase ChKRED20 from *Chryseobacterium* sp. CA49 (Li *et al.*, 2019 ▸). Based on structural alignment, these proteins have very similar folds. However, as was observed with the BoSDR protomer chains (Fig. 4 ▸), the main area where structural features differ is near their respective putative active sites, particularly near the nicotinamide ring of NAD^+^/NADH. Some of these SDRs were crystallized in the presence of the coenzyme, but none of these structures have a potential substrate or substrate analog bound. The structural differences for these enzymes underscore their differing chemistries and provide insight into how their respective substrates access the active site. In these structures, it appears that there is a large solvent cavity leading to the active site that could become more ordered upon substrate binding, as has been observed for other members of the enzyme class (Belfon *et al.*, 2024 ▸). This area is highlighted with a box in Fig. 6 ▸.

## 4. Conclusion

*B. ovis* SDR is a classical SDR that was structurally captured in the presence and absence of the coenzyme NAD^+^. Structural changes are observed in the coenzyme-binding site in the presence and absence of NAD^+^, with the active site becoming more accessible and disordered in the presence of coenzyme. This structure provides intriguing snapshots of the conformational changes in the coenzyme-binding site and the flexibility of the NAD^+^/NADH coenzyme. Specifically, these structures highlight large conformational of the NAD^+^ coenzyme that could be important in the catalytic mechanism. Although the biological substrate of BoSDR is currently unknown, the homology of this enzyme to other SDRs allows us to propose a potential chemical mechanism for an aromatic alcohol. To confirm this mechanism and determine the cellular role of BoSDR, future work will need to be focused on functional assays, including kinetic analyses and small-molecule screening.

## 5. Related literature

The following references are cited in the supporting information for this article: de Beer *et al.* (2014 ▸), Dutta *et al.* (2012 ▸), Perinbaum *et al.* (2017 ▸) and Robert & Gouet (2014 ▸).

## Supplementary Material

PDB reference: oxidoreductase from *Brucella ovis*, 5er6

PDB reference: NAD-bound, 5ha5

Supplementary Figures and Table. DOI: 10.1107/S2053230X25009227/rf5049sup1.pdf

Raw data for PDB entry 5ha5.: https://proteindiffraction.org/search/?q=5ha5

Raw data for PDB entry 5er6.: https://proteindiffraction.org/search/?q=5er6

## Figures and Tables

**Table 1:** Macromolecule-production information

DNA source	Betsy Bricker, USDA
Forward primer	5′-CTCACCACCACCACCACCATATGGAACTTCTGAAAGAAAAGCTCG-3′
Reverse primer	5′-ATCCTATCTTACTCACTTACGCGGCCAGGAAGCCGCCA-3′
Expression vector	pBG1861
Expression host	*E. coli* BL21(DE3)R3 Rosetta
Complete amino-acid sequence of the construct produced	MAHHHHHHMELLKEKLVLVTGAGRGLGAAISSGAAEQGARVILVDIDGTAAKAQADALTAKGFVAEGHALDVTDRDAVAALADDILSRFGGLDVLVNNAGVAGRAAFDQPEAVEVWDRVIGVNLEGAFNVSHALVPALKAAKGNVVHLCSVAGFVSGGSTAGYVVSKGAIRSLTQVMARDLAPHGIRVNAVAPGIMMSEMAVAQLNRPGGTDWFMNRVMMKRIGETSEVVDPVVFLASPMASYITGTILPVDGGFLAA

**Table 2:** Crystallization

	Apo BoSDR	BoSDR–NAD^+^
Method	Vapor diffusion, sitting drop	Vapor diffusion, sitting drop
Temperature (K)	290	290
Protein concentration (mg ml^−1^)	24.8	24.8
Buffer composition of protein solution	20 m*M* HEPES pH 7.0, 0.3 *M* NaCl, 5%(*v*/*v*) glycerol, 1 m*M* TCEP	20 m*M* HEPES pH 7.0, 0.3 *M* NaCl, 5%(*v*/*v*) glycerol, 1 m*M* TCEP
Volume and ratio of drop	0.4 µl protein + 0.4 µl reservoir (1:1)	0.4 µl protein + 0.4 µl reservoir (1:1)
Volume of reservoir (µl)	80	80
Composition of reservoir solution	JCSG+ screen condition H10: 0.1 *M* bis-Tris buffer pH 5.5, 0.2 *M* ammonium acetate, 25%(*w*/*v*) PEG 3350	Morpheus screen condition H2: 10%(*w*/*v*) PEG 8000, 20%(*w*/*v*) ethylene glycol, 0.02 *M* of each amino acid, 0.1 *M* MES/imidazole pH 6.5 supplemented with 5 m*M* NAD^+^
Composition of cryoprotectant	Reservoir solution supplemented with 5%(*v*/*v*) ethylene glycol	Direct cryoprotection from reservoir solution

**Table 3:** Data-collection and processing statistics Values in parentheses are for the outer shell.

	Apo BoSDR (PDB entry 5er6)	BoSDR–NAD^+^ (PDB entry 5ha5)
Diffraction source	21-ID-F, APS	21-ID-F, APS
Wavelength (Å)	0.97856	0.97872
Temperature (K)	100	100
Detector	Rayonix MX-225	Rayonix MX-225
Space group	*P*12_1_1	*P*12_1_1
Crystal-to-detector distance (mm)	140	170
Rotation range per image (°)	1	1
Total rotation range (°)	240	180
Exposure time per image (s)	10	10
*a*, *b*, *c* (Å)	52.01, 94.81, 99.49	52.50, 96.82, 101.90
α, β, γ (°)	90, 99.54, 90	90, 100.75, 90
Mosaicity (°)	0.234	0.271
Resolution range (Å)	50.00–1.55	50.00–1.90
Total No. of reflections	576794	296987
No. of unique reflections	135447	78788
Completeness (%)	98.3 (97.0)	99.9 (99.9)
Multiplicity	4.26 (4.29)	3.77
〈*I*/σ(*I*)〉	12.18 (2.88)	15.52 (2.72)
CC_1/2_ (%)	99.7 (80.3)	99.8 (82.8)
*R*_p.i.m._ (%)	3.62 (24.8)	4.29 (25.4)
Overall *B* factor from Wilson plot (Å^2^)	13	19

**Table 4:** Structure-solution and refinement statistics Values in parentheses are for the outer shell.

	Apo BoSDR (PDB entry 5er6)	BoSDR–NAD^+^(PDB entry 5ha5)
Resolution range (Å)	50.00–1.55 (1.59–1.55)	50.00–1.90 (1.95–1.90)
Completeness (%)	98.3	99.9
σ Cutoff	3.0	3.0
No. of reflections, working set	135447	78757
No. of reflections, test set	2000	2005
Final *R*_cryst_	0.146 (0.214)	0.1519 (0.199)
Final *R*_free_	0.167 (0.233)	0.190 (0.218)
No. of non-H atoms		
Protein	6882	6901
Coenzyme (NAD^+^)	0	176
IMD	0	25
EDO	24	59
ACT	24	0
Water	1135	711
Total	8073	7868
R.m.s. deviations		
Bond lengths (Å)	0.006	0.007
Angles (°)	11	16
Average *B* factors (Å^2^)		
Protein	15	23
Coenzyme (NAD^+^)	N.A.†	26
Other	36 [EDO, ACT]	47 [EDO, IMD]
Water	32	35
Overall	18	24
Ramachandran plot‡		
Most favored (%)	98	97
Allowed (%)	2	2

† N.A., not applicable.

‡ Based on *MolProbity* (Williams *et al.*, 2018 ▸).
