# Supplementary material for: Crystal structure of a short-chain dehydrogenase from Brucella ovis with apo and coenzyme NAD+-bound protomer chains
Source: Acta Crystallogr F Struct Biol Commun. 2025 Nov 11;81(Pt 12):487–94. doi: 10.1107/S2053230X25009227 (PMC12810213; doi:10.1107/S2053230X25009227)
Supplement: Supplementary file 1 [file f-81-00487-sup1.pdf]

STRUCTURAL BIOLOGY COMMUNICATIONS

Volume 81 (2025)

Supporting information for article:

Crystal structure of a short-chain dehydrogenase from *Brucella ovis* with apo and coenzyme NAD$^+$ bound protomer chains

Sean P. Zupko, Amelia T. Konstanty, Steve J. Mayclin, Ryan Choi, Dmitry Serbzhinskiy, Emily Robles, Victoria Moses, Lynn K. Barrett, Wesley C. Van Voorhis, Tom E. Edwards, Peter J. Myler, Andrew T. Torelli, Jarrod B. French and Katherine A. Hicks

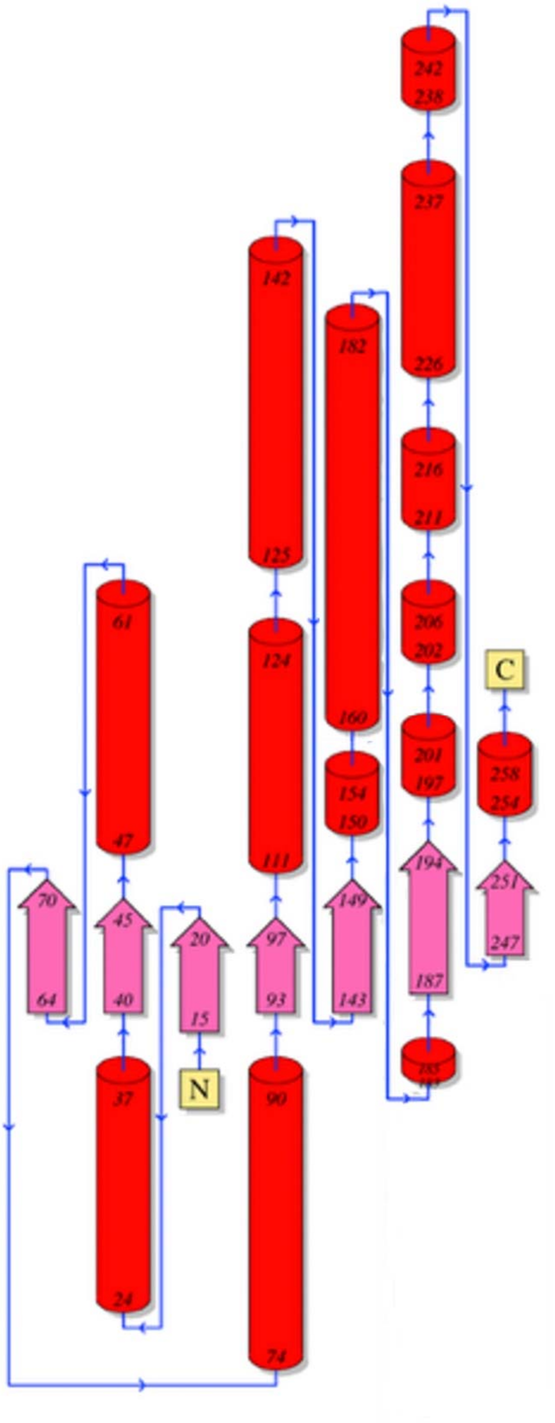

Figure S1 A topology diagram of BoSDR. This figure was made using the *PDBsum* server (de Beer *et al.*, 2014).

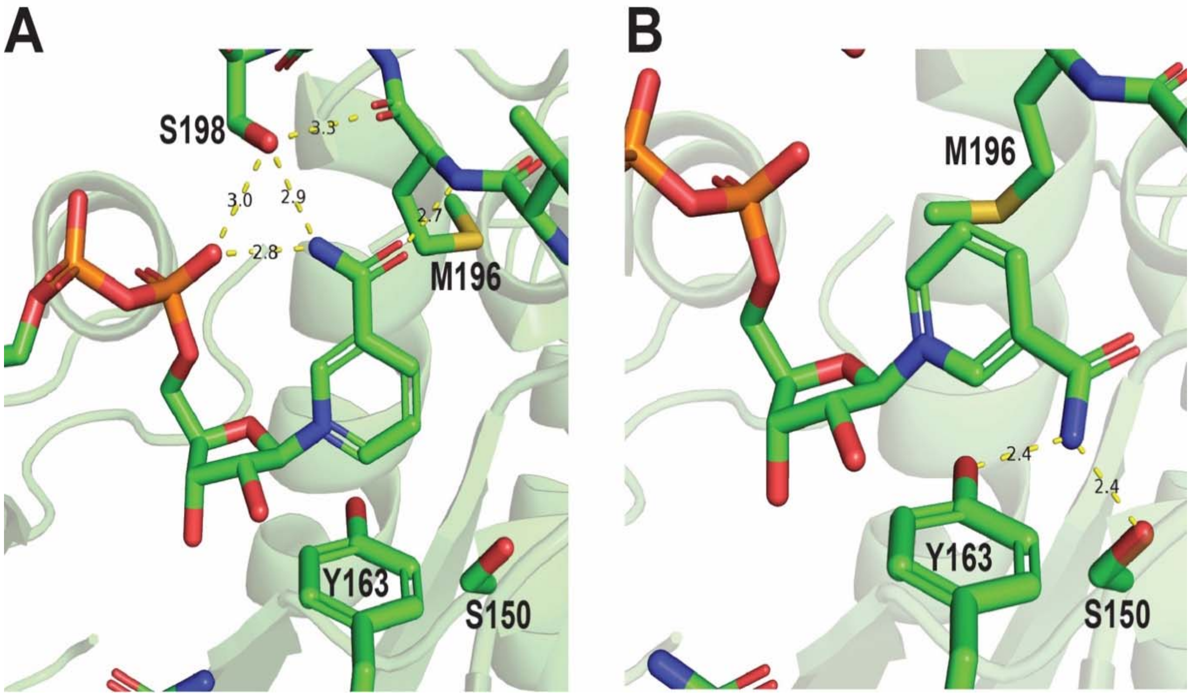

Figure S2 Comparison of the *syn* and *anti* conformations of the NAD^+ coenzyme. Carbon atoms are shown in green. Other heteroatoms are shown using standard coloring. **A**. The coenzyme from BoSDR-NAD^+ (PDB entry 5ha5) chain C is depicted with nearby residues and putative hydrogen bonds depicted as yellow dashed lines. The chain C coenzyme adopts a *syn* conformation that is stabilized by an intramolecular hydrogen bond to the proximal nucleotide phosphate group, which acts as a hydrogen-bond acceptor and allows for the orientation of the exocyclic amide group on the nicotinamide ring to be assigned unambiguously. Serine 198 (not strictly conserved in the sequence alignment, as shown in Figure S4, as in some SDRs it is replaced with the structurally similar threonine) is within a suitable distance to contribute hydrogen bonds to stabilize the nicotinamide ring as well as the proximal coenzyme phosphate. Finally, the carbonyl group on the nicotinamide ring is oriented to accept a hydrogen bond donated by the backbone amide nitrogen atom belonging to Met196. **B**. The chain D coenzyme in PDB entry 5ha5 adopts an *anti* conformation that is stabilized by hydrogen bonds to two conserved active site residues: tyrosine 163, which belongs to the catalytic Y*xxx*K motif, and serine 150, another conserved active-site residue.

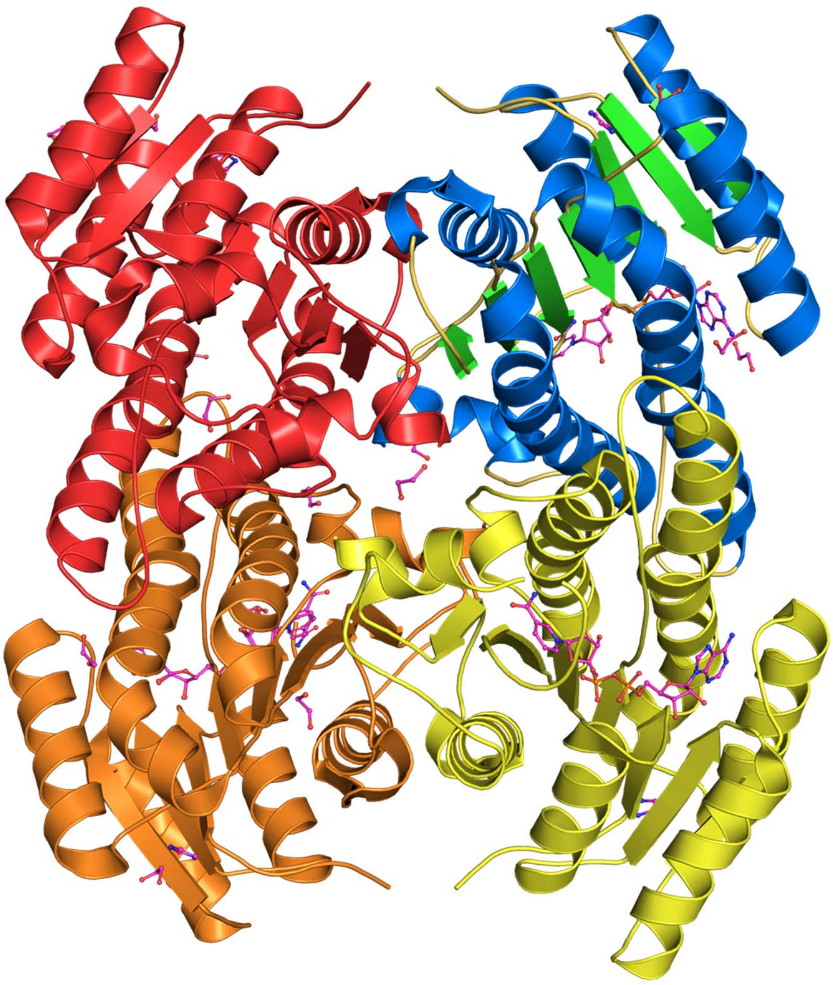

Figure S3 Overall structure of BoSDR highlighting the position of the NAD^+ coenzyme, imidazole and 1,2-ethanediol. The small molecules are shown in ball-and-stick representation with purple carbon atoms. Other heteroatoms are shown using standard coloring. The protomer chains are colored as in Figure 2(*b*).

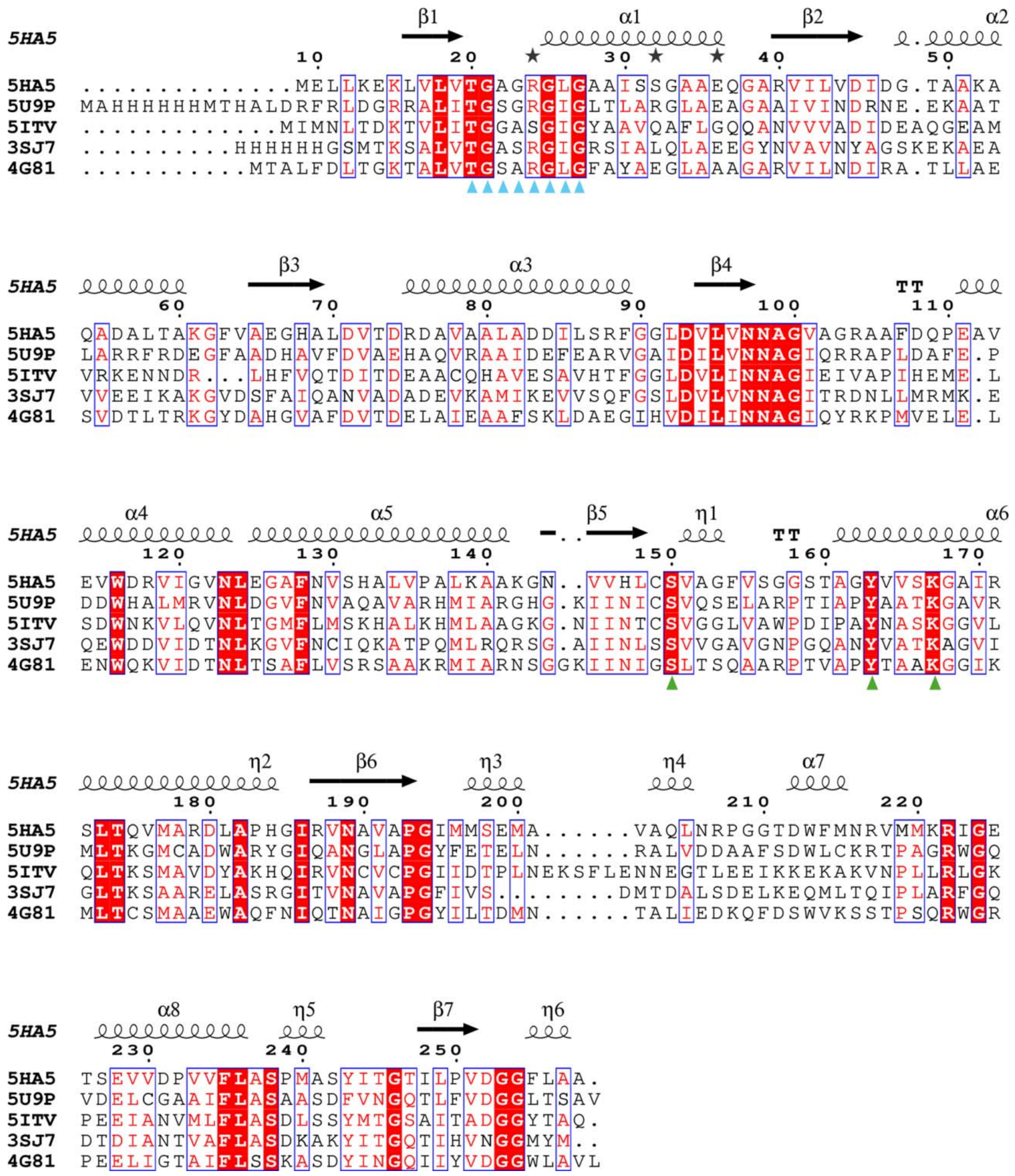

Figure S4 Sequence alignment of BoSDR with other SDRs. A sequence alignment of BoSDR (PDB entry 5ha5/5er6) with gluconate 5-dehydrogenase from *Burkholderia cenocepacia* J2315 (PDB entry 5u9p, 35% sequence identity), *Bacillus subtilis* BacC dihydroanticapsin 7-dehydrogenase (PDB entry 5itv, 38% sequence identity; Perinbam et al., 2017), beta-ketoacetyl-CoA reductase (FabG) from *Staphylococcus aureus* (PDB entry 3sj7, 39% sequence identity; Dutta et al., 2012) and a hexonate dehydrogenase ortholog from *Salmonella enterica* (PDB entry 4g81, 36% sequence identity). The blue triangles show the NAD$^+$ binding site residues, and the green triangles highlight active-site residues. This figure was prepared using the *ESPript* 3.0 server (Robert *et al.*, 2014).

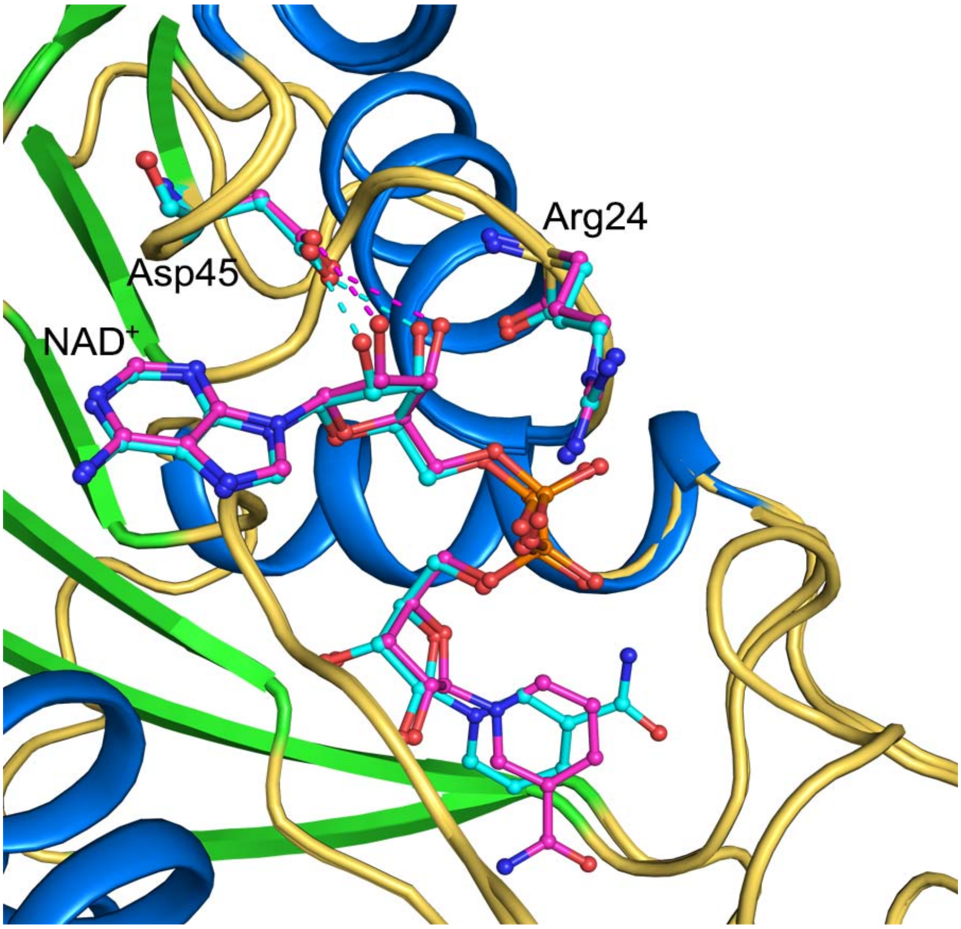

Figure S5 The distance between protein side chains and the hydroxyls on the adenine nucleotide ribose hydroxyls. Here β-sheets are shown in green, α-helices are shown in blue and loops are in yellow. NAD^+ coenzyme is shown in ball-and-stick representation with aqua carbon atoms for chain C and magenta carbon atoms for chain D. Residue carbon atoms have the same coloring. Heteroatoms have standard coloring. Hydrogen bonds between Asp45 and the NAD^+ ribose are highlighted with dashed lines using the same coloring as the carbon atoms. The position of the Arg24 side chain is also highlighted.

Table S1 Structural comparison to other proteins in the Protein Data Bank.

Structural alignment of 5HA5 to all structures in the Protein Data Bank as of June 2025. The top 10 results are shown.

**PDB**[a]	**Z score**[b]	**to**[c]	**%ID**[d]	**Name of Protein**
4NBV	36.4	1.8	33	FabG from *Cupriavidus taiwanensis*
6VSP	36.0	1.6	34	2,3-butanediol dehydrogenase from *Serratia marcescens*
8Y83	35.7	1.7	30	ketoreductase from *Sphingobacterium siyangense* SY1
6IXM	35.7	1.8	30	ketone reductase ChKRED20 from *Chryseobacterium* sp. CA49
4URF	35.6	1.8	32	cyclohexanol dehydrogenase from *Aromatoleum aromaticum* EbN1
3AWD	35.5	1.6	30	putative polyol dehydrogenase from *Gluconobacter oxydans*
8Y7R	35.5	1.7	29	ketoreductase from *Sphingobacterium siyangense* SY1
4JRO	35.4	1.6	33	FabG from *Listeria monocytogenes*
3LQF	35.3	1.6	36	short-chain dehydrogenase Galactitol-Dehydrogenase (GatDH) from *Rhodobacter sphaeroides*
5H5X	35.2	1.6	33	carbonyl reductase from *Streptomyces coelicolor*

[a]Protein Data Bank identifier.
[b]The calculated Z-score.
[c]Root mean square deviation for the alignment.
[d]Percentage sequence identity

References

de Beer, T. A., Berka, K., Thornton, J. M. & Laskowski, R. A. (2014). *Nucleic Acids Res* **42**, D292-296.

Dutta, D., Bhattacharyya, S. & Das, A. K. (2012). *Proteins* **80**, 1250-1257.

Perinbam, K., Balaram, H., Guru Row, T. N. & Gopal, B. (2017). *Protein Eng Des Sel* **30**, 265-272.

Robert, X. & Gouet, P. (2014). *Nucleic Acids Res* **42**, W320-324.
